# In tune with nature: *Wolbachia* does not prevent pre-copula acoustic communication in *Aedes aegypti*

**DOI:** 10.1186/s13071-018-2695-x

**Published:** 2018-02-22

**Authors:** João Silveira Moledo Gesto, Alejandra Saori Araki, Eric Pearce Caragata, Caroline Dantas de Oliveira, Ademir Jesus Martins, Rafaela Vieira Bruno, Luciano Andrade Moreira

**Affiliations:** 1 Mosquitos Vetores: Endossimbiontes e Interação Patógeno-Vetor, Instituto de Pesquisas René Rachou, Fiocruz, Belo Horizonte, MG Brazil; 2 Laboratório de Biologia Molecular de Insetos, Instituto Oswaldo Cruz, Fiocruz, Rio de Janeiro, RJ Brazil; 3 Laboratório de Fisiologia e Controle de Artrópodes Vetores, Instituto Oswaldo Cruz, Fiocruz, Rio de Janeiro, RJ Brazil; 4 Instituto Nacional de Ciência e Tecnologia em Entomologia Molecular (INCT-EM)/CNPq, Rio de Janeiro, Brazil

**Keywords:** *Wolbachia*, *Aedes aegypti*, Mating, Bioacoustics, Harmonic convergence, Fitness

## Abstract

**Background:**

Mosquito-borne diseases are rapidly spreading to vast territories, putting at risk most of the world’s population. A key player in this scenario is *Aedes aegypti*, a hematophagous species which hosts and transmits viruses causing dengue and other serious illnesses. Since vector control strategies relying only on insecticides have proven unsustainable, an alternative method involving the release of *Wolbachia*-harboring individuals has emerged. Its successful implementation vastly depends on how fit the released individuals are in the natural habitat, being able to mate with wild populations and to spread *Wolbachia* to subsequent generations. In mosquitoes, an important aspect of reproductive fitness is the acoustic communication between males and females, which translates to interactions between harmonic frequencies in close proximity flight. This study aimed to characterize the flight tone produced by individuals harboring *Wolbachia*, also evaluating their ability to establish stable acoustic interactions.

**Methods:**

Wild-type (WT) and *Wolbachia*-harboring specimens (*w*MelBr) were thorax-tethered to blunt copper wires and placed at close proximity to sensitive microphones. Wing-beat frequencies (WBFs) were characterized at fundamental and harmonic levels, for both single individuals and couples. Harmonic interactions in homogeneous and heterogeneous couples of WT and *w*MelBr variants were identified, categorized and quantified accordingly.

**Results:**

In tethered ‘solo’ flights, individuals harboring *Wolbachia* developed WBFs, differing slightly, in a sex-dependent way, from those of the WT strain. To test the ability to form harmonic ‘duets’, tethered couples of *w*MelBr and WT individuals were shuffled in different sex pairs and had their flight tones analyzed. All couple types, with WT and/or *w*MelBr individuals, were able to interact acoustically in the frequency range of 1300–1500 Hz, which translates to the convergence between male’s second harmonic and female’s third. No significant differences were found in the proportions of interacting couples between the pair types. Surprisingly, spectrograms also revealed the convergence between alternative harmonic frequencies, inside and outside the species putative hearing threshold.

**Conclusions:**

*Wolbachia* infection leads to small sex-dependent changes on the flight tones of *Ae. aegypti*, but it does not seem to prevent the stereotyped harmonic interaction between males and females. Therefore, when released in the natural habitat to breed with native individuals, *Wolbachia*-harboring individuals shall be fit enough to meet the criteria of acoustically-related mating behavior and promote bacteria dispersion effectively.

**Electronic supplementary material:**

The online version of this article (10.1186/s13071-018-2695-x) contains supplementary material, which is available to authorized users.

## Background

Diseases transmitted by mosquito vectors, with an ever growing human burden, pose a real threat to global public health. The hematophagous species *Aedes aegypti* is a major player in this scenario, being able to host and transmit viruses causing dengue, chikungunya, urban yellow fever and Zika [1–5]. Dengue is the most prevalent arboviral disease, with an estimated 390 million annual infections [1] in over 128 countries [6]. Albeit less frequent, chikungunya and Zika infections are rising and spreading to new territories, including the American continent, where major outbreaks have been reported [7–10]. The remaining burden of vaccine-preventable yellow fever is also alarming and believed to be underestimated in places like Africa, where the annual incidence ranges from 51,000 to 380,000 cases [11].

The escalation of arbovirus infections across the globe is largely attributed to the success of *Ae. aegypti* as a vector [12]. Due to its highly anthropophilic behavior and the ability to quickly adapt to urban environments [13, 14], this species is invading new territories and augmenting its occurrence [15, 16]. With no effective vaccines for dengue, chikungunya or Zika, or even therapeutic drugs to alleviate the diseases’ symptoms, vector control initiatives are the only solution available to fight epidemic outbreaks. Most of these initiatives combine educational approaches, engaging the population to eliminate breeding sites, and the use of insecticides to suppress mosquito populations [12, 17–20]. However, strategies relying on insecticides have proven ineffective and unsustainable for the long term, due to the surge of resistant populations [21, 22].

Recently, an innovative approach using the endosymbiotic bacterium *Wolbachia pipientis* has been successfully implemented to control the transmission of arboviruses by *Ae. aegypti* [23–26]. Naturally present in around 40% of the arthropods [27], *Wolbachia* is an obligatory intracellular symbiont, which promotes its own transmission by manipulating host reproduction through a mechanism known as cytoplasmic incompatibility (CI) [28]. Following an artificial introduction of the bacteria into *Ae. aegypti* [29], a complex host-symbiont association arose and led to an efficient pathogen interference (PI) phenotype, blocking the transmission of dengue, chikungunya and Zika [30, 31].

Fitness costs are also a byproduct of this recent host-symbiont association, thus representing an important concern to release programs of *Wolbachia*-infected lines [26, 29, 32–34]. Depending on the combination between host background and *Wolbachia* strains, higher or lower costs can arise and directly affect the efficacy of which the bacteria spread through native populations [35]. Inducing shorter developmental time and a slightly reduced lifespan, yet keeping strong CI and PI phenotypes, *w*Mel has been the preferred *Wolbachia* strain for control programs [23, 25, 26]. However, some fundamental aspects of reproductive fitness, such as mating behavior, have not been yet assessed for this strain.

Acoustic signals produced during flight play an important role in mosquito mating success. Sexual recognition occurs when males and females, flying within hearing distance, adjust their wing-beat frequencies so that harmonic components can interact. While some species simply converge their fundamental frequencies [36], the great majority of the Culicidae, including *Ae. aegypti*, seem to induce frequency matching at higher harmonics, usually involving male’s second and female’s third components [37–40]. Most importantly, these interactions seem to be important cues for mating success, influencing females’ rejection/acceptance behaviors toward males [41]. For this reason alone, our understanding of mosquito mating behavior is particularly relevant, and should be in taken into consideration when developing control strategies based on the release of *Wolbachia*-harboring lines.

In this report, we characterized the wing-beat frequency of *Wolbachia*-infected *Ae. aegypti* and evaluated their ability to sexually communicate through acoustic signals. To give this work a sense of field application, we chose *w*MelBr as our *Wolbachia*-hosting strain. *w*MelBr is currently being utilized by the ‘Eliminate Dengue’ program in Brazil (http://www.eliminatedengue.com/br) and was obtained by repeated backcrossing (8×) of the original Australian *w*Mel strain with a wild-type (WT) population from Rio de Janeiro.

## Methods

Both *w*MelBr and control (WT) strains were maintained following a standard protocol. Eggs were randomly selected and hatched in distilled water at 28 ± 2 °C. Larvae were sorted into trays filled with 1 l of distilled water and fed a diet of Tetramin® Tropical Tablets (Tetra, Spectrum Brands, Blacksburg, Virginia, USA). Following emergence, adults were immediately sexed (to avoid insemination) and kept at 25 °C on a 10% sucrose diet. Both larvae and adults were reared at 12:12 light-dark cycle.

Experiments were performed during the early morning (ZT0–3; ZT0 = lights ON) and late afternoon hours (around dusk) (ZT9-12; ZT12 = lights OFF), when *Ae. aegypti* is known to be more active [42]. Individuals from the age range of 6 to 10 days were anesthetized on ice and thorax-tethered with super glue to a blunt copper wire. Next, they were positioned at a 2 mm distance to a particle velocity sensitive microphone, located inside a recording apparatus known as INSECTAVOX [43], which was originally developed for acquiring signals from *Drosophila* courtship songs but also proved suitable for mosquito flight tones. Inhibition and stimulation of flight activity was achieved through tarsal contact with a fragment of tissue paper. Recordings of single individuals or couples were performed at 25 ± 1 °C and monitored in real-time, which allowed us to discard samples with erratic wing-beats. For single individuals, recording time was set to 30 s, while for couples there was no time limit, with flight allowed (and stimulated) more than once per recording.

Microphone voltage output was sampled at 44.1 Hz, 16-bit, using Spectrogram v.16 (Visualization Software LLC, Stafford, Virginia, USA). Sound data was stored in wav files and analyzed with Raven Pro v.1.4 (The Cornell Lab of Ornithology, Cornell University, Ithaca, New York, USA; Available from http://www.birds.cornell.edu/raven). Spectrograms were generated following a discrete Fourier Transform (DFT) (every 4096 points or 92.9 ms), Hanning windowing and 50% overlap. Harmonics were measured by manually selecting the spectrograms and applying the ‘center frequency’ algorithm, whose output is the frequency that divides the selection into two frequency intervals of equal energy [44]. Selections spanned 6 s for ‘solo flights’ and 1–4 s for couples with visual indication of convergence (a.k.a. frequency matching), which was corroborated by spectrogram slices with higher resolution. Convergence was not computed, and therefore considered absent in the couple analyzed, if matching frequencies lasted less than 1 s.

## Results

**Fig. 1:**
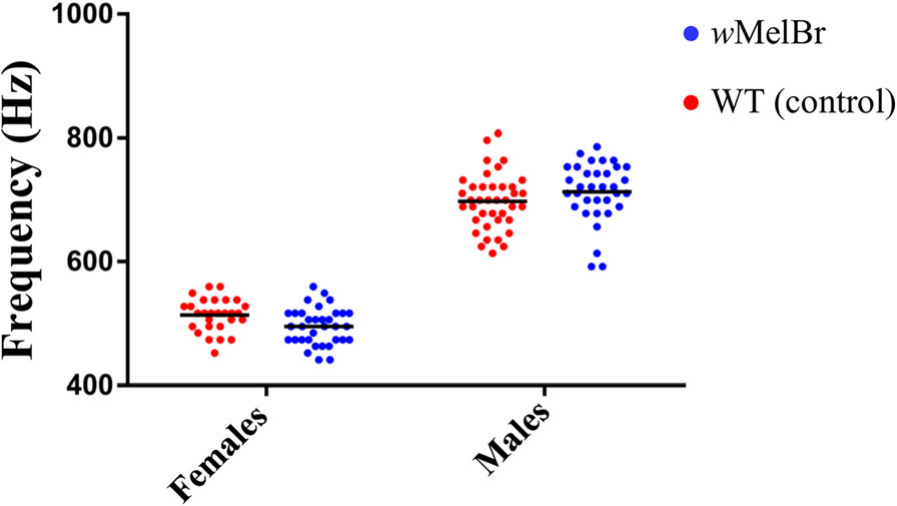
Wing-beat frequencies of *w*MelBr and WT control individuals in ‘solo’ tethered flight. Each dot represents the computed fundamental frequency (i.e. 1st harmonic) for a single adult individual, while the horizontal black lines indicate the mean

To investigate the effect of *Wolbachia* on the modulation of flight tones, we measured the wing-beat frequencies of tethered single individuals, randomly selected from *w*MelBr and WT control strains (Fig. 1). In ‘solo’ flight, the fundamental frequencies (F1) of *w*MelBr males and females were 713.5 ± 8.2 Hz and 495.3 ± 5.1 Hz (mean ± SEM), respectively. The WT control strain showed a similar pattern, with 697.6 ± 7.3 Hz for males and 513.7 ± 5 Hz for females. Statistical analysis of variance (ANOVA) revealed that, while there was a highly significant effect for sex (*F*_(1, 131)_ = 873.4, *P* < 0.0001), there was none for *Wolbachia* (*F*_(1, 131)_ = 0.03728, *P* = 0.8472). Yet, an interesting sex × *Wolbachia* interaction arose (*F*_(1, 131)_ = 6.353, *P* = 0.0129), indicating that the bacteria influence wing-beat frequencies of males and females in opposite ways. Indeed, careful examination of the data plot (Fig. 1) reveals that frequencies for females are slightly lower in *Wolbachia*-harboring individuals, while those for males are higher. Since males are able to detect and orient towards females’ flight tones [45–47], this difference could possibly be an underlying basis for discriminating and selecting sexual partners from each variant. As such, follow-up behaviors were analyzed.

**Fig. 2:**
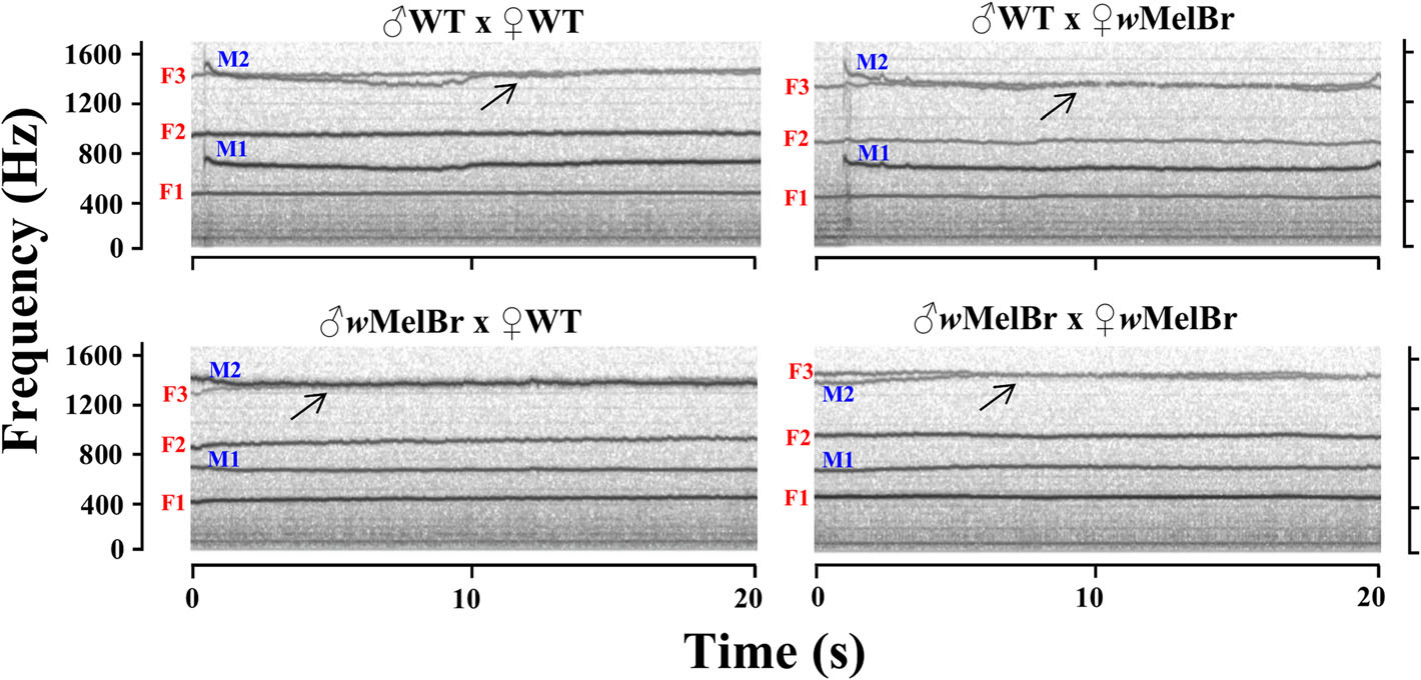
*Wolbachia* does not impair convergence between third harmonic frequencies (F3) for females (red) and second (M2) harmonic frequencies for males (blue) of *Ae. aegypti*. Acoustic interactions were detected in all couple types, formed by WT and/or *w*MelBr individuals. Arrows indicate converging events (aka. Frequency matching). Horizontal and vertical axes represent time (s) and frequency (Hz)

In order to evaluate the effect of *Wolbachia* on the acoustic communication associated with mating, we measured the wing-beat frequencies of tethered couples flying in close proximity. Spectrograms were carefully analyzed for a ‘duet’ formation, which occurs when harmonic frequencies from males and females converge to a common frequency band [37]. Initially, our attention focused on the convergence between females’ third (F3) and males’ second (M2) harmonics, which was found to be an important pre-copula event [37, 41]. To provide a broader picture of any *Wolbachia*-driven effect and re-create the different scenario found when infected individuals are released in the wild, all the possible sex pairs (or couple types) between *w*MelBr and WT were evaluated. Interestingly, our results revealed that all the combinations were able to interact acoustically, suggesting that *Wolbachia* does not prevent this phenomenon (Fig. 2). In strictly WT couples (♂ WT × ♀ WT), 17 out of 35 samples (49%) were able to form ‘duets’ and interact at 1441.5 ± 21.1 Hz, with an interquartile range (IQR) equal to 13.3 ± 1.7 Hz. In couples formed by WT males and *w*MelBr females (♂ WT × ♀ *w*MelBr), duets were observed in 10 out of 28 samples (36%), with converging frequencies of 1357.7 ± 15.8 Hz and IQR of 10.8 ± 0.0 Hz. As for couples of *w*MelBr males and WT females (♂ *w*MelBr × ♀ WT), 6 out 21 samples (29%) were found to converge at 1419.4 ± 34.4 Hz, with IQR of 12.6 ± 1.8 Hz. At last, in strictly *w*MelBr couples (♂ *w*MelBr × ♀ *w*MelBr), 17 out of 32 samples (53%) showed harmonic interaction at 1424.4 ± 17.6 Hz and IQR equal to 13.9 ± 1.2 Hz. One-way ANOVA showed no differences between the means of converging samples from each couple type (*F*_(3, 46)_ = 2.692, *P* = 0.057), indicating that interaction between F3 and M2 occurs in similar frequency ranges. To assess the ability of couples to form these duets, and reveal effects driven by *Wolbachia*, we performed binary logistic regression analyses using SPSS v.17 (IBM). No significant differences were found between strictly WT couples and other types (Wald *χ*^2^ = 4.078, *df* = 3, *P* = 0.253), both in the overall model and in subsequent pairwise comparisons. In addition, it seems that WT males are equally prone to interact with WT or *w*MelBr females (Wald *χ*^2^ = 1.043, *df* = 1, *P* = 0.307), and the reciprocal situation seems to be true for WT females (Wald *χ*^2^ = 2.123, *df* = 1, *P* = 0.145). A similar, non-significant effect, is found when *w*MelBr and WT individuals are challenged by *w*MelBr males (Wald *χ*^2^ = 3.023, *df* = 1, *P* = 0.082) or females (Wald *χ*^2^ = 1.809, *df* = 1, *P* = 0.179). Ultimately, our statistics suggest that F3/M2 interaction is probably not affected by *Wolbachia*.

**Fig. 3:**
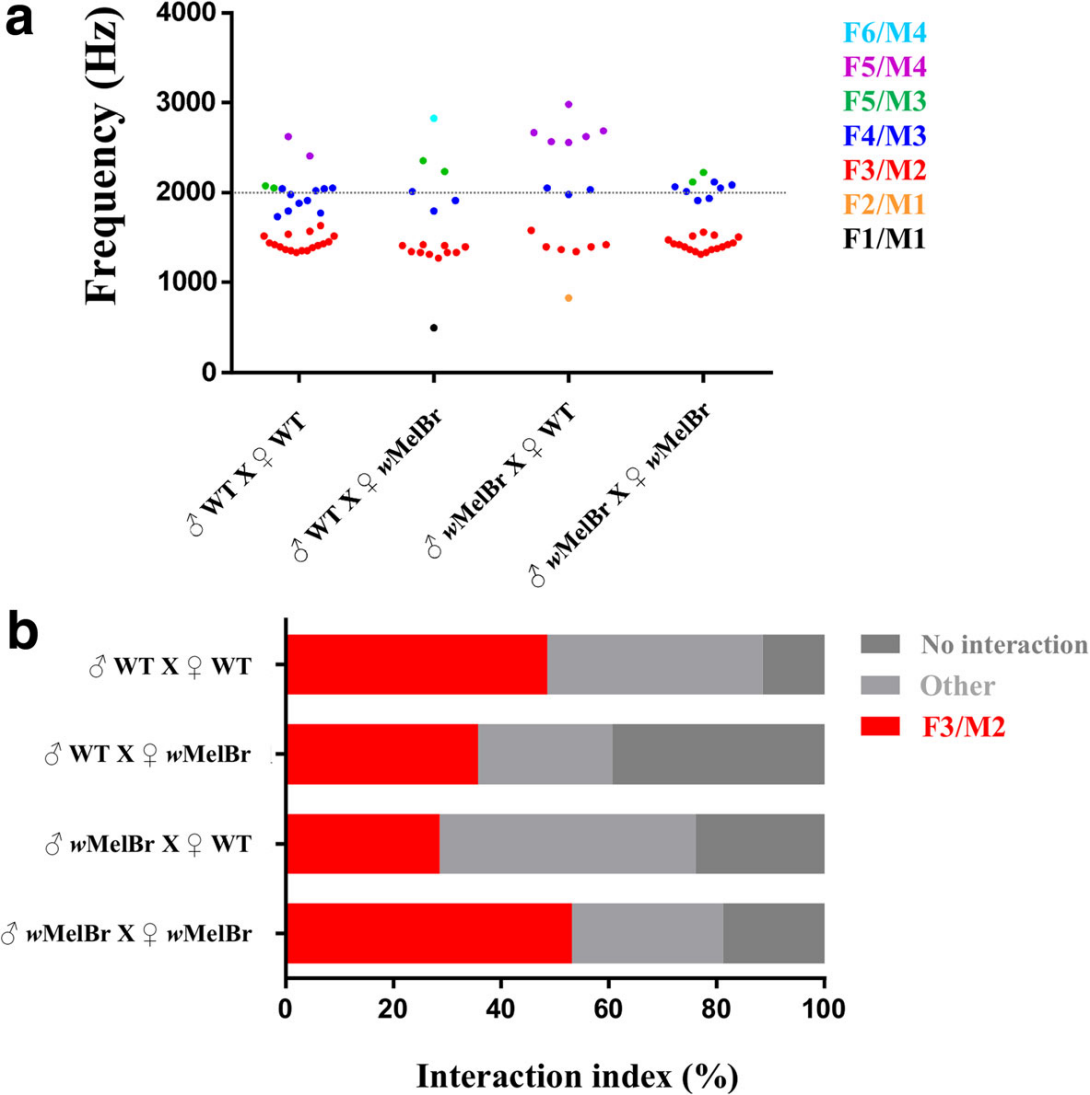
Harmonic convergence of *w*MelBr and WT couples. **a** Distribution of converging samples per couple type. Converging samples were classified by color according to the harmonic frequencies involved in the acoustic interaction, where F/M represent female and male components. The dotted line at 2000 Hz depicts *Ae. aegypti* putative hearing threshold. **b** Relative contribution of F3/M2, alternative (other) and ‘no interaction’ samples

Although most acoustic interactions occurred between F3 and M2, our spectrogram analyses also detected convergence between other harmonic components. In fact, the distribution of all converging samples among couple types revealed a varied array of interactions (Fig. 3a). A fairly common event, for instance, was the interaction between F4 and M3 harmonics (Additional file 1: Figure S1). Less frequent ones included F1/M1, F2/M1, F5/M3, F5/M4 and F6/M4. In terms of percentage, alternative convergence contributes considerably to interaction indexes between couples (Fig. 3b), which could suggest a functional yet unrevealed role. To provide additional insights on the relative contribution of alternative interactions, new statistical analyses were carried out with data sorted in three categories (i.e. ‘F3/M2’, ‘Other’ and ‘No interaction’). No significant effect was found between couple types (*χ*^2^ = 10.49, *df* = 6, *P* = 0.1056), which was further corroborated by pairwise comparisons with multiple corrections assuming a false discovery rate of 0.05. Thus, as it was previously observed, it seems that all couples types show roughly the same ability to interact, even if we consider alternative convergence as a separate category. Another observation that can be drawn from the distribution of converging samples (Fig. 3a) is that heterogeneous combinations (i.e. mixed couples) seem to produce more dispersed data (higher standard deviation) than homogeneous ones (Additional file 2: Table S1). This is particularly evident in couples formed by *w*MelBr males and WT females (SD = 650.7), which should lead to CI and infertile female and, therefore, no offspring. One could speculate that heterogeneous couples, despite being equally able to interact, find some degree of difficulty. Future assays with larger sample sizes, and conditions that better mimic natural mating behavior (e.g. free-flying samples), would be necessary to confirm this effect and take any further conclusions with regards to its functional significance.

## Discussion

By characterizing the wing-beat frequencies of *Wolbachia*-harboring mosquitoes, as well as identifying and analyzing putative acoustic interactions, this work provided novel and important data on the mating behavior of *Ae. aegypti*. First, we revealed that the bacteria affect the wing-beat frequencies of individuals flying ‘solo’, in a sex-dependent fashion. This frequency modulation could be driven by the physical presence of the bacteria either in some sensory organs like the antenna or in flight muscles that mechanically drive wing-beats [23]. Second, we demonstrated that *Wolbachia* does not prevent couples from interacting acoustically by converging harmonic components. As expected, the most common interaction was that between males’ second and females’ third harmonics, forming a well-document duet related to mating success [37, 41]. We found that exclusively *w*MelBr couples were equally prone to interact as WT ones, with roughly the same proportion of samples showing duets. Mixed couples, where *w*MelBr pairs with WT, were also able to interact albeit with apparently lower indexes (not statistically significant). In any case, it seems that *w*MelBr conserves mating behavior, communicating through specific acoustic signals and possibly promoting successful copulas.

Surprisingly, our data also revealed alternative harmonic interactions, occurring both under and above the putative hearing threshold of 2000 Hz [37], with some few events reaching levels close to 3000 Hz (Fig. 3). We hypothesize that these interactions either have no biological significance or constitute important cues for acoustic communication between individuals. If the former is true, than interactions are merely artifacts promoted by odd relationships between two individuals’ wing-beat frequencies. However, if the latter is true, than interactions not yet characterized might contribute to sexual communication by adding an extra level of complexity. This would also imply that electrophysiological recordings have not precisely defined the upper limit of Johnston organ (JO) sensitivity in *Ae. aegypti* and that this species may hear and communicate at frequencies a few hundred hertz higher than previously thought [37]. Alternatively, mosquitoes could be hearing not actual harmonic interactions but an output frequency obtained by the integration of male and female wing-beats [48, 49]. In fact, mosquito hearing mechanisms are currently being revised, due largely to the advent of more comprehensive behavior and physiological audiograms (i.e. tuning curves) [48–50]. It has been reported that the auditory receptors within the JO are unequally represented and individually tuned to different frequency ranges [50]. Other sources of sensory input such as antennal and body hairs may also contribute to a broader range of auditory signals reaching the brain and thus augmenting sensitivity limits [48–50].

In addition, and despite the importance of our findings, recent evidence suggests that acoustic-related mating behavior includes aspects other than harmonic interactions [48, 49]. In species of *Culex* and *Anopheles*, males use acoustic distortion products to detect nearby flying females and to elicit rapid frequency modulation (RFM) of their wing-beats just prior to copula. This phenomenon appears to be essential for mating in both genus and may also exist in *Aedes* spp. Thus, one cannot discard the possibility that *Wolbachia*-harboring and WT males drive distinct RFM in response to their respective female flight tones, provoking a certain degree of assortative mating. In this context, the subtle effect found in the fundamental frequencies of *w*MelBr males and females (Fig. 1) could be differentially translated to acoustic distortions during an RFM response. However, recent evidence suggests that RFM could not to explain the reproductive isolation between sympatric species of the *Anopheles* complex [49]. Future studies are nonetheless necessary to address this point, as to whether the distortion products generated by *Wolbachia*-harboring males could provide any other means for locating and mating with its own variant type.

Regarding the use of *Wolbachia* for vector-borne disease control, our findings are greatly encouraging. The conserved pattern of mating acoustic signaling in individuals carrying the *w*Mel strain shall certainly contribute to their reproductive fitness and facilitate bacterial spread. Once released in the field, these individuals are expected to successfully mate and copulate with wild populations, transmitting the bacteria to the progeny. In subsequent generations, they should be able to breed not only with the wild-type but also with other *w*Mel-harboring individuals, hence keeping the local bacteria load. Corroborating this idea, a mosquito population from Cairns (Australia) still holds *w*Mel infection close to fixation after three years following initial field release [25]. It was also revealed a near perfect maternal transmission rate, as well as intact CI and PI (DENV-blocking), suggesting that *Wolbachia*-host interaction does not significantly change over a brief period of time [25, 51].

It is important to note that *w*Mel’s behavior phenotype may be restricted to this particular strain and genetic background, and should not be extrapolated to others without further investigation. It has already been shown that different *Wolbachia* strains elicit different bacteria-host interactions, hence different host behavioral, metabolic and physiological outcomes [35]. Relevant fitness traits and the particularly important PI phenotype have been measured for some strains, suggesting a delicate trade-off between both. For some strains with very strong PI, such as *w*MelPop, the cost is so high for the host that it struggles to survive in the natural habitat [52, 53]. In this case, it would not be a surprise to find that mating behavior is also disrupted, decreasing reproductive fitness. Conversely, strains like *w*Mel or *w*Alb are less harmful and often associated with milder fitness costs [35], yet still able to drive an effective PI. As our data suggest, mating behavior could be somewhat conserved for strains belonging to this category.

Finally, this work fully supports the current use of *w*Mel-harboring lines to control the spread of dengue and other vector-borne diseases. Without significantly affecting some aspects of the acoustic signaling implied in mating success, as well as other critical traits [23, 26], these lines seem to be fit enough to promote the bacteria invasion in the wild, thus leading to reduced rates of disease transmission and a positive impact on local public health.

## Conclusions

Our acoustic recordings and data analysis suggest that the *w*Mel strain of *Wolbachia* is able to drive small sex-dependent alterations on the fundamental flight tones of *Ae. aegypti*. This effect, however, does not seem to prevent the formation of the ‘so-called’ harmonic duets between males and females. By preserving this important aspect of pre-copulatory behavior, *Wolbachia*-harboring individuals shall be fit enough to acoustically interact and successfully mate with wild variants in field release scenarios, thus contributing to bacteria dispersion and fixation over time.

## Additional files

Additional file 1: **Figure S1.** Alternative acoustic interaction between the fourth harmonic frequency (F4) for a female (red) and the third harmonic frequency (M3) for a male (blue) of *Ae. aegypti*. (TIFF 1525 kb)

Additional file 2: **Table S1.** Descriptive statistics of the couple types. (TIFF 605 kb)
