# Supplementary figures and images for: In tune with nature: Wolbachia does not prevent pre-copula acoustic communication in Aedes aegypti
Source: Parasit Vectors. 2018 Feb 22;11:109. doi: 10.1186/s13071-018-2695-x (PMC5824586; doi:10.1186/s13071-018-2695-x)

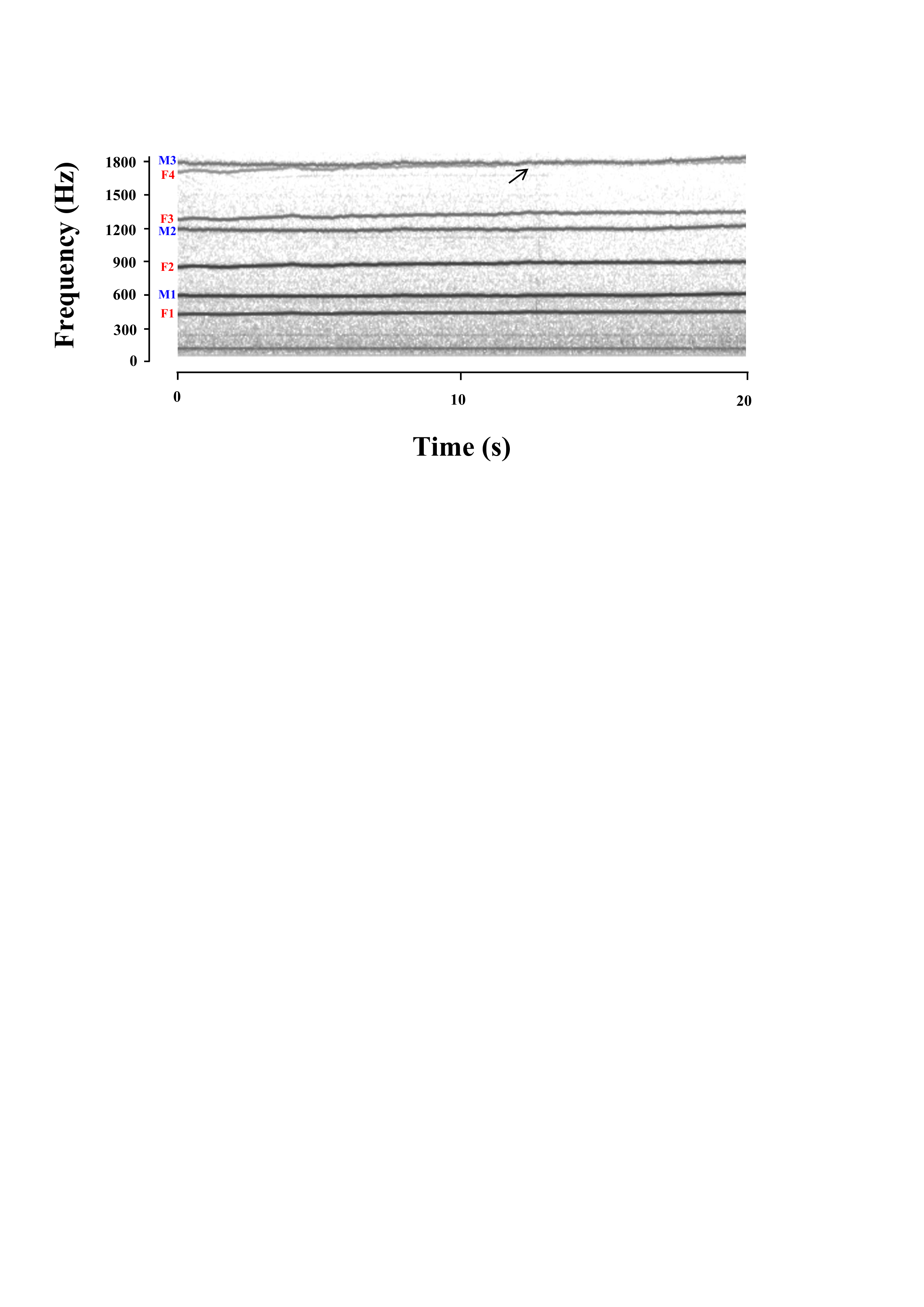

Supplement: Supplementary file 1 — Figure S1. Alternative acoustic interaction between the fourth harmonic frequency (F4) for a female (red) and the third harmonic frequency (M3) for a male (blue) of Ae. aegypti. (TIFF 1525 kb) [file 13071_2018_2695_MOESM1_ESM.tif]

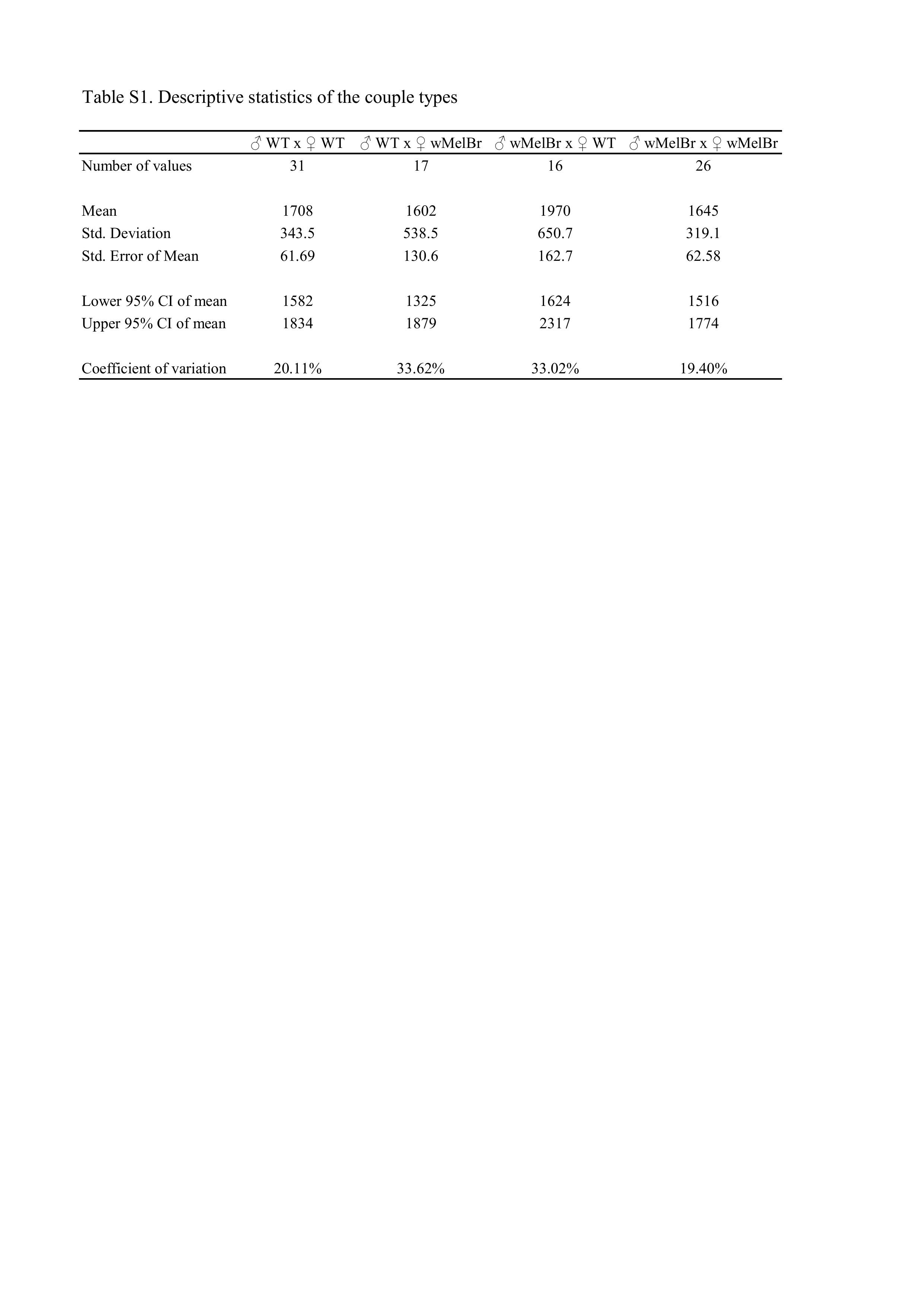

Supplement: Supplementary file 2 — Table S1. Descriptive statistics of the couple types. (TIFF 605 kb) [file 13071_2018_2695_MOESM2_ESM.tif]
